# Rev and Rex proteins of human complex retroviruses function with the MMTV Rem-responsive element

**DOI:** 10.1186/1742-4690-6-10

**Published:** 2009-02-03

**Authors:** Jennifer A Mertz, Mary M Lozano, Jaquelin P Dudley

**Affiliations:** 1 Section of Molecular Genetics and Microbiology and Institute for Cellular and Molecular Biology, the University of Texas at Austin, Austin, TX, USA

## Abstract

**Background:**

Mouse mammary tumor virus (MMTV) encodes the Rem protein, an HIV Rev-like protein that enhances nuclear export of unspliced viral RNA in rodent cells. We have shown that Rem is expressed from a doubly spliced RNA, typical of complex retroviruses. Several recent reports indicate that MMTV can infect human cells, suggesting that MMTV might interact with human retroviruses, such as human immunodeficiency virus (HIV), human T-cell leukemia virus (HTLV), and human endogenous retrovirus type K (HERV-K). In this report, we test whether the export/regulatory proteins of human complex retroviruses will increase expression from vectors containing the Rem-responsive element (RmRE).

**Results:**

MMTV Rem, HIV Rev, and HTLV Rex proteins, but not HERV-K Rec, enhanced expression from an MMTV-based reporter plasmid in human T cells, and this activity was dependent on the RmRE. No RmRE-dependent reporter gene expression was detectable using Rev, Rex, or Rec in HC11 mouse mammary cells. Cell fractionation and RNA quantitation experiments suggested that the regulatory proteins did not affect RNA stability or nuclear export in the MMTV reporter system. Rem had no demonstrable activity on export elements from HIV, HTLV, or HERV-K. Similar to the Rem-specific activity in rodent cells, the RmRE-dependent functions of Rem, Rev, or Rex in human cells were inhibited by a dominant-negative truncated nucleoporin that acts in the Crm1 pathway of RNA and protein export.

**Conclusion:**

These data argue that many retroviral regulatory proteins recognize similar complex RNA structures, which may depend on the presence of cell-type specific proteins. Retroviral protein activity on the RmRE appears to affect a post-export function of the reporter RNA. Our results provide additional evidence that MMTV is a complex retrovirus with the potential for viral interactions in human cells.

## Background

Mouse mammary tumor virus (MMTV) is a betaretrovirus that encodes three accessory and regulatory proteins, a superantigen (Sag) [1-3], a dUTPase [4] and an RNA export protein, Rem [5]. Rem is a 33 kDa protein that is encoded by a doubly spliced mRNA [5,6]. The N-terminal portion of Rem contains nuclear and nucleolar localization signals as well as an arginine-rich motif similar to the RNA export proteins, Rev, Rex, and Rec, produced by the complex retroviruses, human immunodeficiency virus (HIV), human T-cell leukemia virus (HTLV), and human endogenous retrovirus type-K (HERV-K), respectively [5,6]. Our previous data have shown that Rem is larger than other retroviral export proteins due to a unique C-terminus, which negatively regulates Rem-mediated RNA export activity [5]. Negative regulation of MMTV transcription also occurs during viral replication in several cell types [7-10]. MMTV has a complex life cycle that allows transmission through maternal milk to susceptible offspring using dendritic cells as well as B and T cells [11]. Amplification of MMTV in various lymphoid cell types requires virally encoded Sag to effectively transfer virus from lymphocytes to mammary epithelial cells during puberty [12,13]. Both the sophisticated mode of transmission and production of multiple accessory and regulatory proteins imply that MMTV is a complex retrovirus [5].

MMTV may interact with human complex retroviruses. Multiple laboratories previously have reported that MMTV sequences are detectable in human breast cancer or lymphomas, but not most normal tissues, using PCR to amplify one or more regions of the viral genome [14-18]. However, not all studies agree [19,20]. Recent data indicate that MMTV can infect and integrate into chromosomal DNA of cultured human cells [21,22], suggesting that zoonotic infections can occur. Furthermore, MMTV is highly related to HERV-Ks [also known as human MMTV-like proviruses (HMLs)] [23]. Some intact HERV-K/HML-2 proviruses have been described, consistent with their relatively recent acquisition in the human genome, yet none of these proviruses are known to be infectious [24-26]. A number of HERV-Ks are highly expressed in specific tissues [23,27]. In addition, a recent report indicates that antibodies to HERV-K/HML-2 are detectable in the plasma of breast cancer and lymphoma patients, and these titers dropped when the cancers were treated. HERV-K reverse transcriptase activity, viral RNA, processed viral proteins, and virus-like particles also could be detected in patient plasma [28]. Together, these experiments suggest that sporadic MMTV infections of human cells may result in interactions with HERV-Ks or the generation of recombinant infectious viruses.

Prior experiments indicate that HIV Rev and HTLV Rex can activate expression from reporter plasmids containing the HERV-K Rec-responsive element (RcRE) [29]. Because of sequence and organizational similarities between MMTV and HERV-K and the potential for MMTV infection of human cells, we have tested for interactions between heterologous retroviral export proteins and the Rem-responsive element (RmRE) using our previously described reporter vector, pHM*Rluc *[5]. Surprisingly, Rev and Rex, but not Rec, could activate MMTV-based reporter gene expression in human T cells and was dependent on the presence of the RmRE. Cell fractionation experiments followed by RNA quantitation suggested that each of the regulatory proteins, including Rem, did not affect RNA export or stability using an MMTV-based reporter vector. Rem activity was undetectable using heterologous response elements. These results suggest that retroviral export elements recognize similar features of RNA structure and support the idea that MMTV is a complex murine retrovirus that may interact with other retroviruses in human cells.

## Results

**Figure 1:**
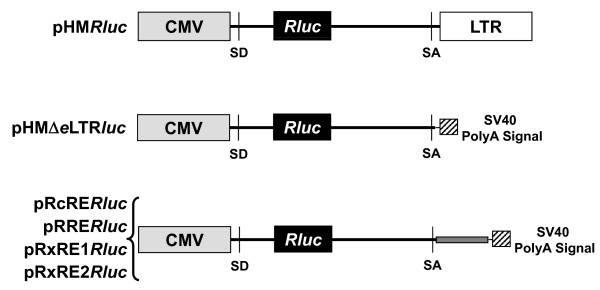
**Structure of plasmids used to determine RNA export activity**. The CMV promoter (gray box) is shown inserted upstream of the 3' end of the MMTV provirus (solid horizontal line). The 3' MMTV LTR is shown by a white box. The *Renilla *luciferase gene (black box) is located between the splice donor (SD) and acceptor (SA) sites. The smaller hatched box indicates the SV40 polyadenylation region. The smaller gray box shows the position for insertion of response elements for other retroviral export proteins within the HMΔeLTR*luc *plasmid.

**Figure 2:**
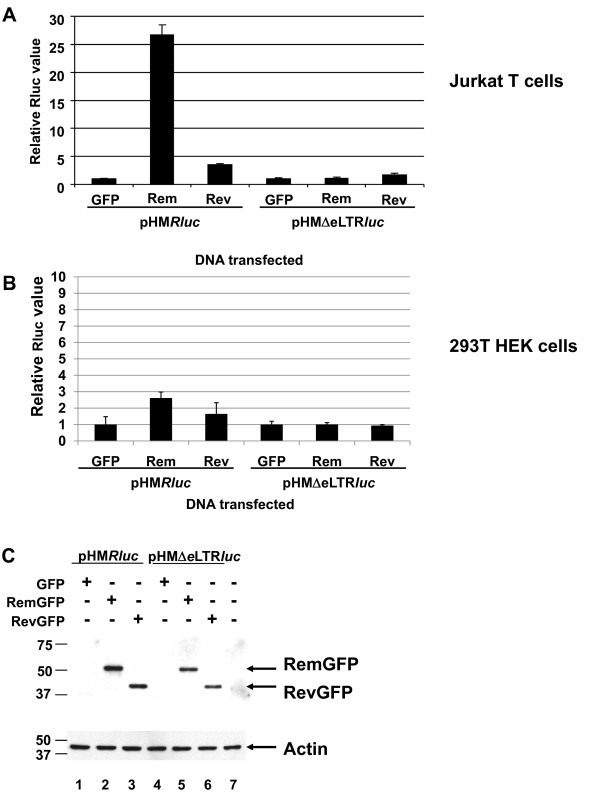
**Activity of HIV-1 Rev on the MMTV RmRE in human cells**. **A**. Reporter activity of RevGFP on the RmRE in Jurkat T cells. Cells were electroporated with pHM*Rluc *or pHMΔeLT*Rluc *(1 μg) with either 20 μg of EGFP, RemGFP or RevGFP expression plasmids. Cytoplasmic extracts were prepared and analyzed for *Renilla *luciferase (Rluc) activity. Average luciferase values for each reporter plasmid in the absence of Rev or Rem have been assigned a value of 1, and the other samples are reported relative to this value after normalization for DNA uptake using a co-transfected pGL3 reporter plasmid expressing firefly luciferase. Standard deviations from the average of triplicate transfections are indicated. **B**. Reporter activity of RevGFP on the RmRE in 293T cells. Cells were transfected using calcium phosphate precipitation of DNA as described in the Methods section. Values are reported as described in panel A. **C**. Western blotting confirms similar expression of Rev and Rem. A Western blot of protein extracts from Jurkat cells is shown. The unfused GFP band is not visible in this portion of the gel. The upper panel shows reactivity with GFP-specific antibody; the lower panel shows equal loading of protein extracts using an actin-specific antibody. Size markers are given in kilodaltons.

To determine if the RNA export proteins from known complex retroviruses function on the MMTV RmRE, we used the reporter vector, pHM*Rluc *(Figure 1) [5]. This vector contains the cytomegalovirus (CMV) promoter upstream of the 3' end of the MMTV genome as well as the *Renilla *luciferase gene between splice donor and acceptor sites [5]. The RmRE appears to span the envelope-3' LTR junction [5,30]. Detection of luciferase activity in transfected cells indicates cytoplasmic export of unspliced transcripts since splicing would remove the reporter gene. Rem expression gave a 25 to 30-fold increase in luciferase expression in Jurkat human T cells relative to cells co-transfected with the reporter plasmid and empty vector (pEGFP), whereas no increase was observed using the reporter lacking a functional RmRE (Figure 2A). Interestingly, Rev expression in Jurkat cells also increased luciferase activity approximately 3-fold compared to control cells expressing only the reporter plasmid (Figure 2A). This result is statistically significant and has been repeated in multiple experiments. Further, Rev-mediated enhancement of reporter activity required the RmRE since deletion of this element eliminated the effect (compare results using pHM*Rluc *or pHMΔeLTR*luc *reporter plasmids) (Figure 2A). The Rev effect on reporter gene expression in Jurkat cells, which is abolished by the Δ3 mutation in the leucine-rich nuclear export sequence (data not shown) [31], is believed to interact with the cellular export protein Crm1 [32,33]. In addition, Rev and Rem-mediated induction of pHM*Rluc *activity was tested by transient transfections of 293T human kidney cells (Figure 2B). Although Rem gave a small, but statistically significant, increase in reporter activity, Rev did not. Both Rem and Rev were expressed as GFP-fusions to allow comparison of the relative expression of these regulatory proteins, and similar amounts were detected using a GFP-specific antibody and Western blotting after transfection of both cell types compared to the actin loading control (Figure 2C and data not shown).

**Figure 3:**
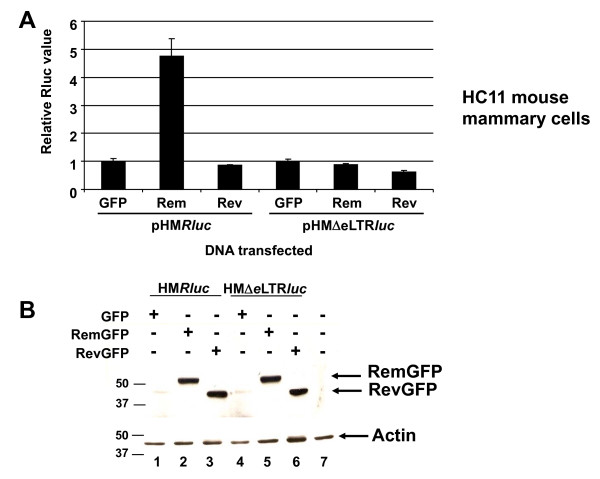
**HIV-1 Rev activity on the MMTV RmRE in mouse mammary cells**. **A**. Reporter activity in HC11 mouse mammary cells. Values are reported as described in Figure 2. **B**. Western blot of Rem and Rev expression in HC11 cells. Similar expression of the GFP-fusion proteins was observed as determined using antibodies specific for GFP (upper panel) or actin (lower panel). Size markers are given in kilodaltons.

The effect of Rev on the MMTV-based luciferase vector also was determined in HC11 mouse mammary cells since breast epithelial cells are permissive for MMTV replication [34]. Rem gave a 4 to 5-fold increase in HC11 cells and was dependent on the presence of the RmRE (Figure 3A), whereas Rev gave no detectable effect in the presence or absence of the response element. Western blots verified protein expression (Figure 3B). Therefore, the heterologous export protein, Rev, appears to function in a cell-type and/or species-specific manner on the MMTV RmRE.

**Figure 4:**
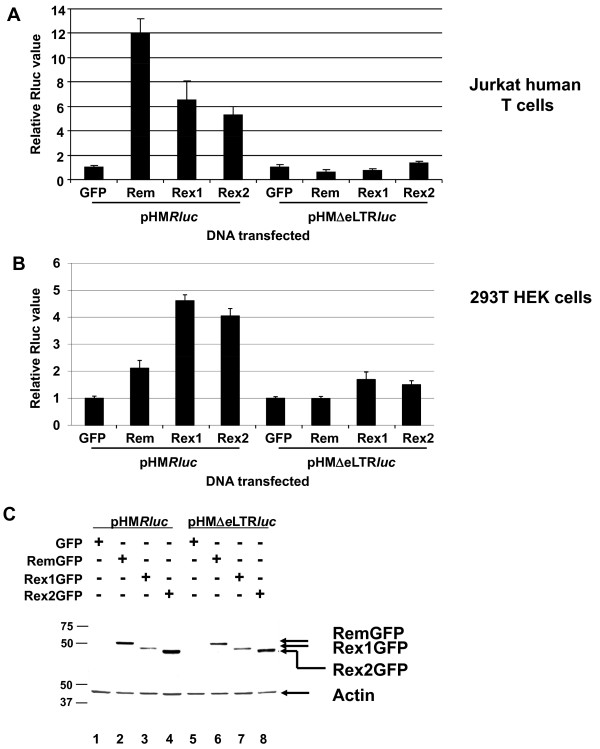
**Activity of HTLV Rex1 and Rex2 on MMTV RmRE-containing reporter plasmids in human cells**. **A**. Reporter activity in Jurkat T cells. **B**. Reporter activity in 293T cells. Values are reported as in Figure 2, except that Rex1GFP or Rex2GFP expression plasmids were used. **C**. Western blotting confirms similar expression of Rem and Rex. Samples from Jurkat transfections are shown and analyzed with antibodies specific for GFP (upper panel) or actin (lower panel). Size markers are given in kilodaltons.

Transfection experiments also were performed after expression of HTLV Rex in the presence of pHM*Rluc *(Figure 4). Rex1 and Rex2 from HTLV-1 and -2, respectively, stimulated luciferase activity in Jurkat cells in a RmRE-dependent manner, although the magnitude of the effect (ca. 5 to 7-fold) was greater than that observed for Rev (Figure 4A). Rex also was tested for stimulation of reporter gene expression in human kidney epithelial cells (293T) [35]. Rem and Rex stimulated reporter expression 2-fold and 4-fold, respectively, and was dependent on the RmRE (Figure 4B). Western blotting showed similar expression of these proteins (Figure 4C and data not shown). Thus, the stimulation was dependent on the presence of the RmRE in human cells.

**Figure 5:**
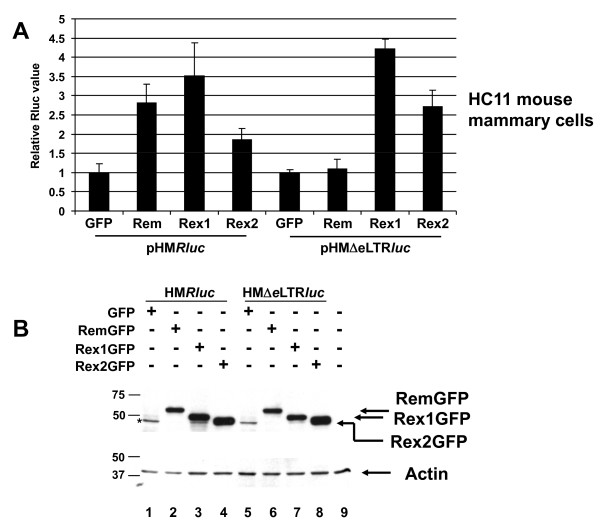
**HTLV Rex1 and 2 activity on the MMTV RmRE in mouse mammary cells**. **A**. Reporter activity in HC11 mouse mammary cells. Values are reported as in Figure 2, except that Rex1GFP and Rex2GFP expression plasmids were used. **B**. Western blots of extracts from Rex and Rem-transfected HC11 cells. A GFP-related band is observed in this blot (asterisk; lanes 1 and 5), but the major band is not visible in this portion of the gel (upper panel). Similar levels of Rem, Rex1 and Rex2 fusion proteins are observed using the GFP-specific antibody. Incubation with an actin-specific antibody revealed similar protein loading in each lane (lower panel). Size markers are given in kilodaltons.

No RmRE-dependent effect of Rex1 or 2 was observed in mouse HC11 epithelial cells (Figure 5A), although Rex has been shown to function in mouse fibroblast cells [36]. However, 2- to 4-fold increases were observed using both the pHM*Rluc *and pHMΔ*e*LTR*luc *vectors, which we attribute to cell-type-specific effects of Rex since greatly diminished activity was observed in human cells after RmRE deletion (Figure 4A). Furthermore, vectors that substituted the RmRE with the RxRE gave a 7- to 9-fold increase in luciferase expression in HC11 cells, which was dependent on Rex, but not Rem (see Figure 8). Expression of GFP-fusion proteins was equivalent in mouse and human cells as determined by Western blotting (Figures 4C and 5B). Therefore, mouse epithelial cells may lack cell-type specific proteins that allow Rex function on the MMTV RmRE. Like Rev, these results suggest that the enhancement of reporter activity by Rex is species or cell-type specific, whereas the effect of Rem is not.

**Figure 6:**
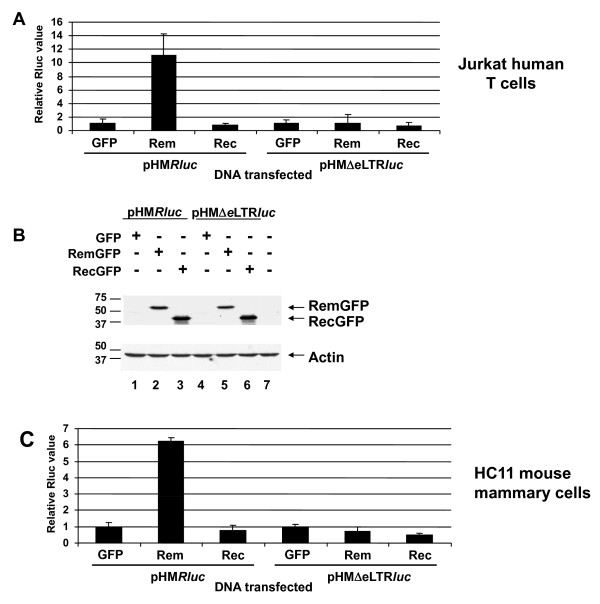
**Activity of HERV-K Rec on a reporter plasmid containing the MMTV RmRE in human and mouse cells**. **A**. Reporter activity in Jurkat T cells. **B**. Western blotting of Rem and Rec expression in Jurkat cells. Results using antibodies specific for GFP (upper panel) and actin (lower panel) are shown. Size markers are given in kilodaltons. **C**. Reporter activity in HC11 mouse mammary cells. Values in panels A and C are reported as in Figure 2, except that a RecGFP expression plasmid was used.

Although HIV and HTLV are only distantly related to MMTV, the human endogenous retrovirus type K (HERV-K) has sequence similarity to MMTV and is a betaretrovirus that encodes the RNA export protein, Rec [37]. Both HERV-K Rec and MMTV Rem are translated from the same open reading frame as their respective envelope signal peptides, but Rec lacks the extensive autoregulatory region found in the Rem C-terminus [5,29]. Rec expression in either Jurkat (Figure 6A) or HC11 (Figure 6C) cells failed to enhance the basal luciferase activity of pHM*Rluc *despite demonstrable expression of the GFP-fusion proteins (Figure 6B and data not shown). Together, these results indicated that Rev and Rex, but not Rec, could stimulate expression of unspliced RNA containing the RmRE.

**Figure 7:**
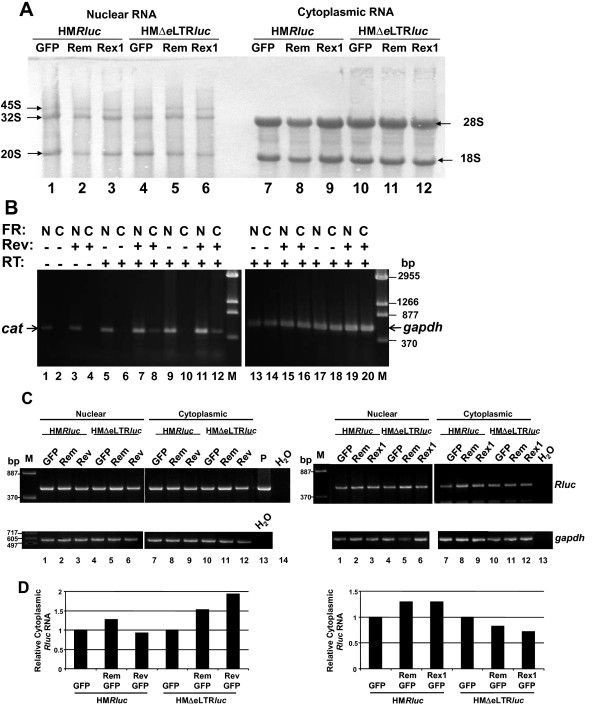
**Fractionation experiments indicate that Rev and Rex have little effect on export or stability of unspliced RmRE-containing reporter transcripts**. **A**. Integrity of cytoplasmic and nuclear fractions obtained from transfected Jurkat cells. Jurkat cells were subjected to transfection by electroporation and, after 48 hr, cells were fractionated. Fractions were used for RNA extraction and subjected to Northern blotting prior to staining with methylene blue and photography. The nuclear ribosomal precursors (arrows on the left) and cytoplasmic mature ribosomal RNAs (arrows on the right) are indicated. **B**. Semi-quantitative RT-PCRs of fractionated RNA obtained from Jurkat cells transfected with the HIV-based reporter vector pDM128. Cells were co-transfected with pDM128 and either pEGFPN3 control vector (no Rev) or RevGFP (Rev) expression plasmids as indicated by minus or plus signs. After 48 hr, cells were fractionated, and RNA samples were extracted and subjected to RT-PCRs using primers for the *cat *gene or glyceraldehyde-3-phosphate dehydrogenase (*gapdh*). Fractions (FR) used for RNA extraction are indicated as nuclear (N) or cytoplasmic (C). PCRs performed in the absence of reverse transcriptase (RT) are indicated (lanes 1–4) (equivalent to 2 μl of a diluted cDNA reaction). Either 2 μl (lanes 5–8 and 13–16) or 4 μl (lanes 9–12 and 17–20) of the diluted cDNAs were used for RT-PCRs as indicated in Methods to show that the reactions were performed in the linear range. Samples were analyzed on a 1.5% agarose gel using either 5 (*gapdh*) or 15 μl (*cat*) of the 50 μl reaction. Markers (M) are given in basepairs (bp). **C**. Semi-quantitative RT-PCR assays of the MMTV-based reporter plasmids in the presence or absence of RemGFP, RevGFP, and RexGFP. Semi-quantitative RT-PCRs were performed using RNA extracted from transfected Jurkat cells and primers specific for the *Renilla *luciferase (*Rluc*) or *gapdh *genes. Left and right panels show results of two different transfection experiments. M = DNA markers (in bp); P = HM*Rluc *plasmid positive control; H_2_0 = PCR without added cDNA. **D**. Quantitation of RT-PCR results from cytoplasmic fractions of cells transfected with the MMTV-based reporter plasmid. Stained RT-PCR products from panel C were quantitated using ImageJ software and normalized for RNA amounts and integrity using *gapdh *expression. The normalized RNA levels obtained from each reporter plasmid (in the presence of the control EGFP expression vector only) were assigned values of 1, and the other samples have been reported relative to these values. These results are representative of at least three different transfection experiments.

To determine whether the HIV Rev and HTLV Rex proteins enhanced luciferase expression from the MMTV-based reporter vector through increases in RNA export or another mechanism, transfected Jurkat cells were subjected to cellular fractionation. After different detergent concentrations were tested to optimize the integrity of the fractions, nuclear and cytoplasmic RNAs were obtained, and samples were subjected to Northern blotting and staining to confirm the isolation of intact RNA and absence of rRNA precursors in the cytoplasmic fractions (Figure 7A). Subsequently, Jurkat cells were subjected to electroporation with the HIV-based reporter vector, pDM128, in the presence and absence of a RevGFP expression plasmid. Transfected cells were used to obtain nuclear and cytoplasmic fractions. RNA samples from these fractions then were subjected to semi-quantitative reverse transcription (RT)-PCRs using primers specific for the *cat *reporter gene (Figure 7B). As expected [38-40], increased cytoplasmic levels of unspliced RNA containing the chloramphenicol acetyl transferase (*cat*) gene in pDM128 are observed in the presence of Rev (compare lanes 6 and 8 as well as 10 and 12 with two different amounts of cDNA). Although controls indicated that the nuclear fractions in this experiment were contaminated with DNA (Figure 7B, lanes 1 and 3), the absence of contaminating DNA in the cytoplasmic fractions further substantiated the integrity of the cellular fractionations. RT-PCRs using *gapdh*-specific primers confirmed similar levels of intact RNA (lanes 13–20). PCR conditions did not appear to be saturated since higher product levels could be observed for the more abundant *gapdh *mRNA than for reporter transcripts.

Additional experiments then were performed in Jurkat cells using the MMTV-based reporter vector (pHM*Rluc*) and co-transfected expression vectors for GFP-tagged Rem, Rev, or Rex. RNAs from cytoplasmic and nuclear fractions were treated with DNase I and subjected to semi-quantitative RT-PCRs using primers specific for the *Renilla *luciferase gene or *gapdh *[5] (Figure 7C). PCRs without added reverse transcriptase showed that DNA contamination was absent (data not shown). Cytoplasmic RNA levels then were quantitated using ImageJ software after normalization for *gapdh *expression. Unexpectedly, these experiments indicated that Rem, Rev, or Rex had little effect on the levels of RNA in the nucleus or cytoplasm in Jurkat cells, suggesting that these proteins do not affect intron-containing transcript stability or export (Figure 7D).

**Figure 8:**
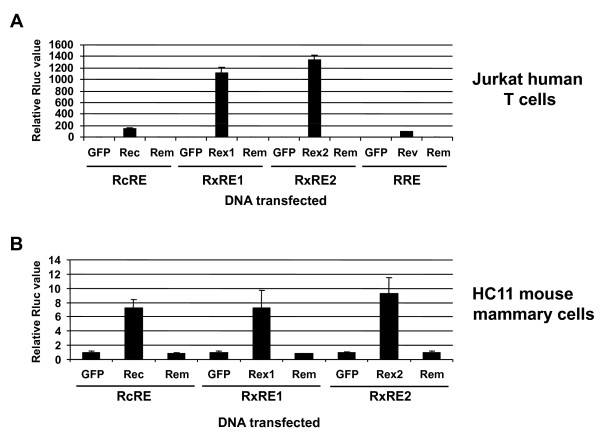
**Rem lacks activity on heterologous RNA export elements**. **A**. Rem activity on heterologous export elements in Jurkat T cells. Cells were transfected with reporter plasmids containing the indicated response elements described in Figure 1 with or without expression vectors for retroviral export proteins. **B**. Rem activity on heterologous export elements in HC11 mouse mammary cells. Relative luciferase values in both panels are reported as described in Figure 2.

We also tested the ability of Rem to affect expression using heterologous response elements in both Jurkat and HC11 cells (Figure 8). The response elements from HERV-K, HTLV-1, HTLV-2, and HIV were cloned into pHMΔeLTR*luc*, which lacks a functional RmRE, resulting in the reporter plasmids pRcRE*Rluc*, pRxRE1*Rluc*, pRxRE2*Rluc*, and pRRE*Rluc*, respectively (Figure 1). As expected, each of these plasmids gave significantly increased (100 to 1300-fold) luciferase activity in human Jurkat T cells in the presence of their homologous export protein compared to the activity of the reporter plasmid in the presence of unfused GFP (Figure 8A). Similar transfections also were performed in HC11 mouse mammary cells (Figure 8B). Since Rev had no demonstrable activity on the RmRE in HC11 cells, Rev and the pRRE*Rluc *construct were not tested in these cells. Rec, Rex1, and Rex2 showed increased reporter activity with their homologous response elements in HC11 mammary cells, but function was substantially reduced compared to that observed in Jurkat cells (Figure 8A). Further, Rem failed to enhance expression from any of the tested response elements in either cell line. Western blotting with GFP-specific antibody confirmed similar levels of each export protein (data not shown). These results suggest that the RmRE secondary and/or tertiary structure does not duplicate other retroviral export elements. However, the ability of Rem, Rev and Rex to function on the RmRE in human cells suggests common features of RNA recognition.

**Figure 9:**
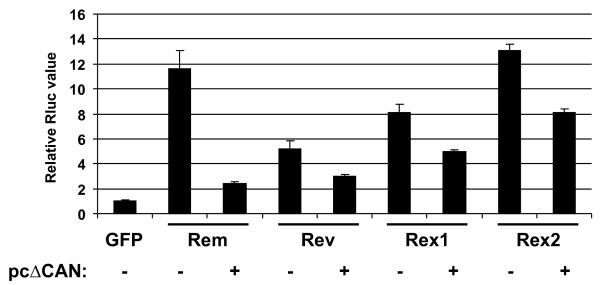
**Rev and Rex use the Crm1 pathway to enhance expression of RmRE-containing RNAs**. Jurkat cells were electroporated with 1 μg of pHM*Rluc *and 1 μg of pGL3 control firefly luciferase plasmid. The EGFP, RemGFP, RevGFP, Rex1GFP or Rex2GFP expression plasmids (10 μg) were added as indicated with or without 20 μg of the plasmid expressing the dominant-negative nucleoporin (pcΔCAN) [42]. All samples were adjusted to the same concentration of DNA with empty vector (pBC12/CMV) prior to transfection. Luciferase activity was determined as described in Figure 2. *Renilla *luciferase values were normalized for DNA uptake using firefly luciferase activity, and the pHM*Rluc *sample cotransfected with EGFP was assigned a relative value of 1.

We have previously shown that Crm1 is required for Rem function in HC11 mouse mammary cells [5]. Rev and Rex also use Crm1 for the export of intron-containing homologous RNAs [32,41]. To test the involvement of Crm1 in human cells, we tested whether a dominant-negative nucleoporin involved in the Crm1 export pathway (ΔCAN) [42] would affect the increased luciferase activity mediated by Rem, Rev, or Rex in Jurkat T cells. The dominant-negative protein gave a statistically significant suppression of Rem, Rev, Rex1 and Rex2 activation of the HM*Rluc *vector, although the greatest effect was observed with Rem (Figure 9). Suppression by the ΔCAN mutant did not appear to be toxic for the cells under these conditions (data not shown). Furthermore, our previous data indicated that overexpression of a dominant-negative Tap/NXF1 mutant (TapA17) had no effect on Rem-induced reporter activity in HC11 mouse cells [5]. Similarly, TapA17 overexpression in human Jurkat cells did not affect Rev, Rex, or Rem-mediated stimulation of HM*Rluc *activity (not shown). Neither ΔCAN nor TapA17 had a dramatic effect on reporter activity in the absence of a regulatory protein (not shown). Together with previous results, these data suggest that enhancement of reporter activity by Rem requires Crm1 and that the regulatory proteins facilitate a post-export function that depends on the RmRE.

## Discussion

Our previous experiments have shown that MMTV Rem functions in nuclear export of unspliced viral RNA in rodent cells [5]. In this manuscript, we have shown that Rem functions in human cell lines. Our results also indicate that Rev and Rex can increase reporter gene expression by interaction with the MMTV RmRE in human Jurkat T cells (Figures 2 and 4). Rex also could function on the RmRE in 293T HEK cells. Prior data indicate that some retroviral export proteins function on heterologous retroviral RNAs. For example, Rex can bind and function on both the RRE and the RxRE [43,44]. However, the interaction is not reciprocal since Rev cannot act on the RxRE [43]. In this respect, the RmRE is quite permissive since it is required for enhancement of luciferase activity by Rem, Rev and Rex in human T cells. No effect of Rem was observed on the HIV and HTLV response elements (Figure 8). Surprisingly, the Rec export protein from the human retrovirus most closely related to MMTV, HERV-K/HML, had no effect on expression from the MMTV RmRE (Figure 6). Further, no effect of Rem was observed on the RcRE, although both HIV Rev and HTLV Rex have been reported to increase expression from the HERV-K response element [29]. However, polymorphisms have been observed in different HERV-K proviruses [29], and it is possible that other RcRE variants might function with MMTV Rem or that other Rec variants may be functional on the RmRE. Given that the regulatory proteins require formation of specific secondary structures rather than a simple primary sequence [29,45-48], Rec also may need secondary or tertiary structures not found in the RmRE.

The effect of Rev and Rex on the MMTV RmRE appears to be specific in human cells by several criteria. First, increases in reporter gene activity that were dependent on the RmRE were only observed in human cells with Rex or Rev. Although different results have been reported [36,49], Rev appears to function in both mouse and human cell lines using vectors with a design similar to that of pHM*Rluc*, but based on the 3' end of the HIV genome [50]. Rex also has been reported to function in both human and mouse cells [36], although Rev and Rex have primarily been tested in fibroblasts [36,50], which are not natural target cells for HIV, HTLV or MMTV. Second, a Rev mutant defective in the nuclear export sequence gave no specific effect in the pHM*Rluc *assay, similar to the effect observed with RRE-containing vectors [31]. Third, a dominant-negative mutant nucleoporin in the Crm1 pathway inhibited Rem, Rev, and Rex activation of reporter expression through the RmRE in human cells. Rem previously has been shown to require Crm1 in rodent cells [5], whereas Rev and Rex use Crm1 in human cells [33,41]. Fourth, no effect of Rec was observed on the RmRE in either mouse or human cells. Fifth, insertion of different response elements in the pHM*Rluc *vector yielded the expected increases in luciferase activity after expression of the homologous export protein. These results indicate that the MMTV-based vector allows the activity of other response elements and that each of the GFP-fusion proteins is functional (Figure 8). Although we may have lowered the sensitivity for detection of regulatory protein function in mouse cells by testing fusion proteins, Western blotting using an antibody that recognizes all of the fusion proteins allowed us to verify that similar amounts of each protein were expressed in transfection assays. Prior experiments by Dangerfield et al. suggest that Rev can bind to the MMTV LTR and stimulate luciferase expression from constructs containing the MMTV LTR in monkey cells [51]. Our studies differ significantly since their data were obtained by insertion of MMTV sequences into an HIV-based vector, and the ability of Rem to function on heterologous response elements was not determined. Furthermore, only the MMTV LTR, which lacks a portion of the RmRE [30] (our unpublished data), was present in the HIV vector [51]. Thus, our data argue for a specific effect of HIV and HTLV regulatory proteins on the MMTV RmRE in human cells.

Previous experiments from our laboratory have shown that human Jurkat T cells can produce mature MMTV particles after transfection of a cloned provirus, and these particles are infectious for mice [52,53]. Consistent with this observation, our current data indicate that Rem can function in human cells. The reports of MMTV infection of human cells and detection of MMTV sequences in breast cancers and lymphomas [14-18] appear to be feasible since most steps of viral replication occur in human cells. Cell entry would provide the primary barrier to infection [54]. Although human cell infections appear to be inefficient and infrequent, certain cell types may have an additional entry receptor, which is dependent on cellular activation or differentiation state. The ability of Rev and Rex to function on the MMTV RmRE in human T cells suggests that rare interactions of these viruses could occur.

Rev is known to have multiple functions, including enhancement of RNA encapsidation of HIV and SIV-based vectors [55]. Our previous results indicated that export of unspliced MMTV RNA and Gag expression from a transfected MMTV provirus requires Rem in rat fibroblast cells [5]; encapsidation was not measured. The reporter vector pHM*Rluc *is based on the 3' end of the MMTV provirus and has been shown to be responsive to Rem only in the presence of the RmRE in rat, mouse, and human cells [5] (this study). Further, the use of the *Renilla *luciferase gene in the vector provides both a sensitive and highly quantitative assay, which is difficult to achieve using RNA fractionation experiments and Northern blotting. Rev/RRE interactions also have been shown to affect Gag trafficking and HIV assembly, and it has been suggested that export elements facilitate "marking" of RNAs in the nucleus for particular events in the cytosol [56]. Our experiments show that Rev and Rex function through Crm1 on pHM*Rluc *(Figure 9). Nevertheless, cell fractionation experiments with the pHM*Rluc *vector indicate that the regulatory proteins primarily lacked effects on nuclear RNA export and RNA stability (Figure 7). Since effects on cytosolic RNA levels and Gag production were clearly demonstrable using an MMTV proviral clone with a transposon insertion into the *rem *coding sequence [5], our results with the pHM*Rluc *vector suggest that different sequence elements at the 5' end of the full-length MMTV RNA allow additional effects of Rem on RNA stability and/or export.

Published experiments indicate a wide variability (0 to 10-fold) in Rev function on RNA export [55,57-60]. Suboptimal splicing appears necessary to allow the accumulation of genomic HIV RNA and the export effects of Rev [61]. Efficiency of splicing of full-length MMTV RNA versus pHM*Rluc *vector RNA appears to be an unlikely explanation for differences in observed nuclear export. The splice donor and acceptor sites found in pHM*Rluc *are those normally used to generate either the *rem *or *sag *fully spliced mRNAs, and the low abundance of these RNAs in MMTV-infected cells [6,62] suggests that splicing at these sites is suboptimal compared to those used to produce MMTV *env *RNAs from genomic RNAs. Rev also appears to overcome effects of several cis-acting repressive sequences, including sequences within *gag-pol *[63,64] as well as *env *sequences that overlap with the RRE [65,66]. The repressive sequences in HIV *gag-pol *appear to be AU-rich, and mutation led to increased steady state RNA levels [64]. The pHM*Rluc *vector lacks *gag-pol *sequences (Figure 1), but our previous work with Rem-deficient MMTV genomic clones was consistent with defective RNA export, rather than a stabilization effect.

The cell fractionation data with pHM*Rluc *(Figure 7) and MMTV genomic length RNA [5] argue that Rem has multiple functions, including both export and post-export activities. Rev and Rex have been reported to function at the level of translation [59,67,68]. Specific cis-acting elements found in the RU5 and *gag *regions of several retroviruses appear to affect translation [69-73], but such sequences are absent in the pHM*Rluc *vector. Since the post-export function of Rem with pHM*Rluc *is sensitive to competition with a Crm1-binding site on Nup214 (ΔCAN) (Figure 9), it is possible Crm1 dictates Rem protein export independent of the vector RNA. Nevertheless, Rem binding to the 3' RmRE, perhaps in the cytoplasm or after binding of a cellular protein in the cytoplasm, may promote a post-export step, such as translation. Rem binding through sequence elements at the 5' end of the MMTV RNA may increase Crm1-dependent export, but such 5' elements may not be necessary for detection of the post-export activity of the pHM*Rluc *vector. Our current data indicate that the RmRE maps to the junction of the envelope gene and the 3' LTR using deletion analysis with the pHM*Rluc *vector and co-transfection of a Rem expression vector (see below). Interestingly, these results suggest that all MMTV mRNAs contain the 3' RmRE, unlike the RRE, which would be removed from completely spliced HIV mRNAs, such as those encoding Tat, Nef, and Rev [74]. Previously published data indicate that export of unspliced genomic MMTV RNA, but not partially spliced envelope RNA, is leptomycin B and, by implication, Crm1-dependent [6]. Such experiments suggest that only unspliced MMTV RNA is selectively exported. Therefore, it is possible that the MMTV genome contains two RmREs, one at the 5' end of viral RNA present only in unspliced RNA and a second element at the 3' end present in all MMTV RNAs. The 3' element may facilitate translation of all mRNAs, whereas the 5' element would specifically facilitate nuclear export of genomic RNA. Cell-type specific effects also may occur. Characterization of the molecular mechanisms of Rem function will require further investigation.

Both the pHM*Rluc *vector and MMTV genomic RNAs contain a RmRE that spans the envelope-LTR junction [30] (Mertz et al., in preparation). Published data indicate that retroviral export/regulatory proteins bind to complex RNA structures that have multiple stems and loops [29,48,75,76]. Rev and Rec appear to bind to RNA stems with a bulge, and recognition of heterologous elements may not occur through the same primary sequence as the homologous protein [29]. Our current data using RmRE susceptibility to several RNases is consistent with a complex structure containing multiple stems and bulges, which encompasses a region of ca. 500 bases (Mertz et al., in preparation) rather than the single stem with multiple bulges previously proposed [30]. The export of unspliced retroviral RNA is known to require specific cellular proteins, such as hnRNPs and Sam68 [77,78], and binding of these cellular proteins may determine the cell-type specificity observed in our experiments. Since retroviral export/regulatory proteins recognize certain RNA secondary structures [48,79], one or more of these proteins may bind to and function on specific cellular RNAs as reported for Rex [80].

RNA-binding proteins appear to regulate several steps following transcription, leading to coordinated regulation of cellular RNAs with related functions called RNA regulons [81]. MMTV replication in the mouse requires several different cell types, including lymphocytes and mammary epithelial cells [34]. We previously have shown that MMTV replication is controlled at the transcriptional level during mammary gland development coordinately with several milk-specific genes [82,83]. Therefore, post-transcriptional control of MMTV expression also may be modulated by Rem depending on the cell type and state of differentiation. Our results provide additional evidence that MMTV is a murine complex retrovirus with the potential to interact with human retroviruses [5].

## Methods

### Cell lines and transfections

Jurkat human T lymphoma cells were maintained in RPMI media supplemented with 5% fetal calf serum (FCS), gentamicin sulfate (50 μg/ml), penicillin (100 U/ml) and streptomycin (50 μg/ml). HC11 normal murine mammary epithelial cells were maintained in RPMI supplemented with 10% FCS, gentamicin sulfate (50 μg/ml), penicillin (100 U/ml), streptomycin (50 μg/ml), insulin (0.5 μg/ml) and epidermal growth factor (0.5 μg/ml). The 293T human embryonic kidney cells were grown as previously described [84] in Dulbecco's modified Eagle's medium containing 7.5% fetal bovine serum and antibiotics.

Jurkat cells were transfected by electroporation using a BTX ECM600 instrument. Cells (1 × 10^7^) were mixed with the appropriate plasmid DNA in a volume of 400 μl RPMI medium prior to electroporation in 4 mm gap cuvettes (260 V, 1050 μF and 720 ohms). Transfected cells then were incubated at 37°C in complete medium and harvested two days after transfection for Western blotting and reporter assays. HC11 cells also were transfected by electroporation using a BTX electroporator. Cells (1 × 10^7^) were mixed with the appropriate plasmid DNA in a volume of 200 μl of RPMI prior to electroporation in 2 mm gap cuvettes at 140 V, 1750 μF, and 72 ohms. The 293T cells were transfected essentially as described [35] by the calcium phosphate method. On the day before transfection, 5 × 10^5 ^cells were added to each well of a 6-well plate, and DNA (total of 6 μg) in 0.25 M CaCl_2 _(100 μl) was added dropwise to 100 μl of 2× HBS (280 mM NaCl, 10 mM KCl, 1.5 mM disodium phosphate, 12 mM dextrose and 50 mM HEPES, pH 7.05) with vortexing. The precipitate was allowed to form at room temperature for 10 to 15 minutes, and the solution was added dropwise to the cells in growth medium. Cells then were incubated at 37°C from 4 to 8 hours, the medium was removed, and cells were washed in phosphate-buffered saline prior to replacement with fresh growth medium. Transfected cells were harvested after two days and assayed for reporter gene levels and protein expression. All transfections were performed in triplicate and contained the same total amounts of plasmid DNA. A constant amount of pGL3 control containing the firefly luciferase gene was included in each transfection to normalize for any differences in DNA uptake. Some experiments also tested for DNA uptake after determination of the percentage of cells expressing a GFP control vector using FACS analysis. No significant differences were observed using either of the two methods. All reported experiments were repeated at least twice with similar results.

### Plasmid constructs

The RemGFP, HM*Rluc *and HMΔ*e*LTR*luc *plasmids have been described [5]. The plasmid EGFPN3 was obtained from Clontech, and pGL3-Control plasmid was obtained from Promega. The expression plasmid for the Δ3 mutation in the Rev nuclear export sequence was received from Dr. Tom Hope. The pcΔCAN (dominant-negative Nup214) and pcTapA17 (dominant-negative Tap/NXF1) expression plasmids were kindly provided by Dr. Bryan Cullen (Duke University). The empty vector pBC12/CMV was obtained by excision of the ΔCAN cDNA from pcΔCAN. The pRRE*Rluc *plasmid was constructed by insertion of the HIV-1 RRE, amplified from the pDM128 vector (provided by Dr. Tom Hope), into an engineered *Sca*I site downstream of the splice acceptor site and upstream of the SV40 poly(A) signal in HMΔ*e*LTR*luc*. The plasmids RxRE1*Rluc *and RxRE2*Rluc *were generated by amplification of RxRE1 and RxRE2 from pcgagRxREI and pcgagRxRE2, respectively (provided by Dr. Pat Green) and insertion into an engineered *ScaI *site downstream of the splice acceptor site and upstream of the SV40 poly(A) signal in pHMΔeLTR*luc*. The RcRE*Rluc *plasmid was made by amplification of the RcRE from pJY76 (provided by Dr. Bryan Cullen) and insertion into an engineered *Sca*I site downstream of the splice acceptor site and upstream of the SV40 poly(A) signal in pHMΔeLTRl*uc*. RevGFP, Rex1GFP, Rex2GFP and RecGFP were generated by cloning of the individual cDNAs in-frame with a C-terminal GFP tag in the vector EGFPN3.

### Reporter assays

Luciferase assays were performed using the dual-luciferase reporter assay system (Promega) to quantitate both *Renilla *and firefly luciferase activities [85].

### Northern blotting and RT-PCRs

RNA was extracted from transfected Jurkat cells as described previously [86], except that the lysis buffer (10 mM Tris-HCl, pH 8.0, 140 mM NaCl, 1.5 mM MgCl_2_, 20% glycerol) contained 0.1% NP-40 rather than 0.5% NP-40. Lysis buffer was supplemented with 10 mM vanadyl ribonucleoside complexes (New England Biolabs) to inhibit ribonucleases prior to use. Cells were mixed using a vortex mixer, examined for lysis by microscopy, and nuclei were pelleted by centrifugation (300 × g for 5 minutes at 4°C). The supernatant (cytoplasmic fraction) was removed and again subjected to centrifugation (1,200 × g for 5 minutes). The cytoplasmic fraction was then subjected to centrifugation at 8,000 × g for 5 minutes at 4°C. The nuclear pellet was washed once with lysis buffer, and the supernatant containing residual cytoplasm discarded. Nuclear samples were processed in Tri-Reagent (2 M guanidine isothiocyanate, 12.5 mM sodium citrate, pH 7.0, 0.25% Sarkosyl, 0.05 M 2-mercaptoethanol, 0.2 M sodium acetate, pH 5.2, and 50% water-saturated phenol, pH 7.5), whereas Tri-Reagent LS (Molecular Research Center, Inc.) was used for cytoplasmic fractions. Samples then were processed as described by the manufacturer. RNAs were precipitated using ethanol, washed in 70% ethanol, and precipitates were collected by centrifugation at 10,000 × g for 30 minutes at 4°C. DNA was removed after precipitation of high-molecular-weight RNA in 3 M sodium acetate [87], pellets were washed in 70% ethanol, and the quantity of the RNA was determined by absorbance readings at 260 nm. Procedures for Northern blotting using formaldehyde-containing agarose gels have been described [88]. To test for the integrity of the cellular fractionation, each lane of the gel contained 10 μg of fractionated RNA prior to electrophoresis and transfer to Hybond N+ nylon membranes in 0.15 M sodium citrate and 1.5 M NaCl. RNA samples then were cross-linked to the membrane using UV light and stained with methylene blue prior to photography.

Fractionated RNAs from transfected cells also were used for RT-PCRs. Each RNA sample (1 μg) was digested with 1 U of DNase I (amplification grade, Invitrogen) in the presence of 0.5 μl of RNaseOUT (Invitrogen) ribonuclease inhibitor for 15 minutes at room temperature. The reaction was terminated by the addition of EDTA to 2.5 mM and incubation for 10 minutes at 65°C. The treated RNAs then were incubated with 50 pmol oligo(dT)_17 _primer and 1 mM deoxyribonucleoside triphosphates for 5 minutes at 65°C and then quickly cooled on ice for 5 minutes. Subsequently, first-strand buffer (Invitrogen) was added in the presence of 10 mM DTT, 20 U RNaseOUT, and 200 U of Moloney murine leukemia virus reverse transcriptase (Invitrogen) in a 20 μl reaction. Samples were incubated for 50 minutes at 37°C and then terminated by heating at 70°C for 15 minutes. PCRs were performed using 1 μl of the cDNA reaction, 25 pmol of each primer, 0.2 mM deoxyribonucleoside triphosphates, 20 mM Tris-HCl pH 8.55, 2.5 mM MgCl_2_, 16 mM ammonium sulfate, 100 ug/ml BSA, and 2.5 U of KlenTaq (Sigma Aldrich) in a reaction volume of 50 μl. Samples were subjected to 3 minutes at 94°C for 3 minutes followed by 35 cycles consisting of incubations at 94°C for 1 minute, 50°C for 45 seconds, and 72°C for 45 seconds. The primers used to detect unspliced RNAs containing the *Renilla *luciferase gene were *Rluc*1409(+) (5' GAT TGG GGT GCT TGT TTG G 3') and *Rluc*1904(-) (5' TTC CCA TTT CAT CAG GTG C 3'). Primers for *gapdh *have been described [5]. Similar reactions for *cat*-specific transcripts contained 5 μg RNA and 1.5 U DNase I, and 3.5 μg of the treated RNA was used to make cDNA in 20 μl and then diluted two-fold. Either 2 μl or 4 μl of the diluted cDNA was used in 50 μl PCRs containing *cat *primers [186(+) (5' TCT TGC CCG CCT GAT GAA TGC 3') and 653(-) (5' CCG CCC TGC CAC TCA TCG CAG 3')] and REDTaq mix (Sigma-Aldrich). PCR samples were analyzed on 1.5 or 2% agarose gels and stained with ethidium bromide prior to photography.

### Antibodies and Western blotting

Western blot assays were performed essentially as described previously [5]. Transfected cells were harvested to obtain whole-cell extracts by addition of one volume of 250 mM Tris-HCl, pH 6.8, 20% glycerol, 2% sodium dodecyl sulfate (SDS), 5% 2-mercaptoethanol, and 0.2% bromophenol blue to cells in one volume of phosphate-buffered saline (PBS) followed by boiling for 5 minutes. Proteins were resolved on 10 or 12% polyacrylamide gels containing 1% SDS and transferred to a nitrocellulose membrane. Membranes were blocked with 5% milk in Tris-buffered saline Tween 20 (TBST; 20 mM Tris-HCl, pH 7.6, 137 mM NaCl, and 0.1% Tween 20) for 1 hour. The primary antibody was diluted in TBST containing 5% milk and incubated with the membrane for 1 hour followed by three washes in TBST for 5 to 10 minutes each. The horseradish peroxidase-conjugated secondary antibody was diluted in TBST containing 1% milk and incubated with the membrane for 45 minutes prior to three additional 5 to 10 minute washes. All steps were performed at 25°C with shaking. Western Lightning enhanced chemiluminescent reagent (Perkin-Elmer) was used to detect antibody binding. Monoclonal antibodies specific for actin (Calbiochem) or GFP (Becton Dickinson) were used at a dilution of 1:10,000 or 1:8000, respectively.

## Competing interests

The authors declare that they have no competing interests.

## Authors' contributions

JAM, MML, and JPD performed the experiments. JAM and JPD conceived the experiments, and all authors participated in writing the manuscript. JAM, MML and JPD have approved the manuscript.
